# Algorithmic Classification of Psychiatric Disorder–Related Spontaneous Communication Using Large Language Model Embeddings: Algorithm Development and Validation

**DOI:** 10.2196/67369

**Published:** 2025-05-30

**Authors:** Ryan Allen Shewcraft, John Schwarz, Mariann Micsinai Balan

**Affiliations:** 1 Department of Global Biometrics & Data Sciences, Bristol Myers Squibb, 3551 Lawrenceville Rd, Lawrence Township, NY, 08648, United States, 1 800-332-2056

**Keywords:** psychiatric disorders, large language models, speech, language, spontaneous communication, social media, LLM, communication, algorithm, emotion, schizophrenia, borderline personality disorder, BPD, depression, attention-deficit/hyperactivity disorder, ADHD, anxiety, posttraumatic stress disorder, PTSD, bipolar disorder, assessment, monitoring

## Abstract

**Background:**

Language, which is a crucial element of human communication, is influenced by the complex interplay between thoughts, emotions, and experiences. Psychiatric disorders have an impact on cognitive and emotional processes, which in turn affect the content and way individuals with these disorders communicate using language. The recent rapid advancements in large language models (LLMs) suggest that leveraging them for quantitative analysis of language usage has the potential to become a useful method for providing objective measures in diagnosing and monitoring psychiatric conditions by analyzing language patterns.

**Objective:**

This study aims to explore the use of LLMs in analyzing spontaneous communication to differentiate between various psychiatric disorders. We seek to show that the latent LLM embedding space identifies distinct linguistic markers that can be used to classify spontaneous communication from 7 different psychiatric disorders.

**Methods:**

We used embeddings from the 7 billion parameter Generative Representational Instruction Tuning Language Model to analyze more than 37,000 posts from subreddits dedicated to seven common conditions: schizophrenia, borderline personality disorder (BPD), depression, attention-deficit/hyperactivity disorder (ADHD), anxiety, posttraumatic stress disorder (PTSD) and bipolar disorder. A cross-validated multiclass Extreme Gradient Boosting classifier was trained on these embeddings to predict the origin subreddit for each post. Performance was evaluated using metrics such as precision, recall, *F*_1-_score, and area under the receiver operating characteristic curve (AUC). In addition, we used Uniform Manifold Approximation and Projection dimensionality reduction to visualize relationships in language between these psychiatric disorders.

**Results:**

The 10-fold cross-validated Extreme Gradient Boosting classifier achieved a support-weighted average precision, recall, *F*_1_, and accuracy score of 0.73, 0.73, 0.73, and 0.73, respectively. In one-versus-rest tasks, individual category AUCs ranged from 0.89 to 0.97, with a microaverage AUC of 0.95. ADHD posts were classified with the highest AUC of 0.97, indicating distinct linguistic features, while BPD posts had the lowest AUC of 0.89, suggesting greater linguistic overlap with other conditions. Consistent with the classifier results, the ADHD posts have a more visually distinct cluster in the Uniform Manifold Approximation and Projection projects, while BPD overlaps with depression, anxiety, and schizophrenia. Comparisons with other state-of-the-art embedding methods, such as OpenAI’s text-embedding-3-small (AUC=0.94) and sentence-bidirectional encoder representations from transformers (AUC=0.86), demonstrated superior performance of the Generative Representational Instruction Tuning Language Model-7B model.

**Conclusions:**

This study introduces an innovative use of LLMs in psychiatry, showcasing their potential to objectively examine language use for distinguishing between different psychiatric disorders. The findings highlight the capability of LLMs to offer valuable insights into the linguistic patterns unique to various conditions, paving the way for more efficient, patient-focused diagnostic and monitoring strategies. Future research should aim to validate these results with clinically confirmed populations and investigate the implications of comorbidity and spectrum disorders.

## Introduction

Psychiatric disorders encompass a diverse range of conditions affecting an individual’s thoughts, emotions, and behaviors. These disorders are characterized by complex and heterogeneous symptomatology, making it difficult to establish precise diagnostic criteria and monitor disease progression over time [1]. While standardized clinical interviews and questionnaires are commonly used, they rely on subjective assessments and can be time-consuming or insensitive to subtle changes, potentially leading to misdiagnosis and delayed intervention [2] .

Language, as a fundamental aspect of human communication, reflects the intricate interplay between thoughts, emotions, and experiences. Quantitative analysis of language usage has emerged as a valuable tool for providing objective measures for diagnosing and differentiating between different psychiatric disorders. Studies have demonstrated that language-based features, such as syntactic complexity, semantic coherence, and emotional valence, can serve as reliable markers for differentiating between psychiatric disorders. For instance, individuals with schizophrenia often exhibit disturbances in their speech patterns, characterized by disorganized syntax and impaired semantic coherence [3]. Similarly, individuals with borderline personality disorder (BPD) have higher levels of overall expressive language impairment, as well as decreased syntactic and lexical complexity [4].

Furthermore, quantitative analysis of language usage can aid in tracking disease progression and treatment response. Longitudinal studies have demonstrated that changes in linguistic patterns over time can be indicative of disease progression and treatment outcomes. For example, alterations in language usage have been correlated with changes in current depression symptoms [5]. In addition, “tentativeness,” as measured by a higher degree of uncertainty, is correlated with quantitative levels of symptoms of anxiety measured by the Generalized Anxiety Disorder 7-item (GAD-7) scale [6]. These findings underscore the potential of quantitative language analysis as a sensitive and objective measure for monitoring disease trajectories and treatment efficacy.

Recent advancements in large language models (LLMs) have opened up exciting possibilities for quantitative assessment of neurological diseases [7,8]. Due to their transformer architecture, LLMs project strings of text (sentences, paragraphs, etc) onto a high-dimensional embedding space that represents the semantic and syntactic relationships between words and phrases. In this embedding space, linguistically similar texts are geometrically co-located. Therefore, we hypothesize that, given the differences in patterns of speech by individuals across psychiatric disorders, spontaneous use of language will occupy diagnosis-specific subspaces in the LLM embedding space. To test this hypothesis, we specifically focus on using embeddings derived from the Generative Representational Instruction Tuning Language Model (GritLM-7B) LLM [9] to classify posts originating from subreddits dedicated to six common conditions: schizophrenia, BPD, depression, attention-deficit/hyperactivity disorder (ADHD), anxiety, posttraumatic stress disorder, and bipolar disorder (BD) [10].

This investigation represents an innovative application of LLMs in the field of psychiatric disorders. By using LLM embeddings to analyze the spontaneous use of language in online discussion data, we aim to provide a proof-of-concept for an LLM-based framework to encourage further development of more objective, efficient, and patient-centered strategies for assessment, monitoring, and research.

## Methods

### Data Collection

The dataset we used in this study was obtained from the publicly available dataset provided by Low et al [10], which can be accessed on Zendodo [11]. The dataset consists of posts from seven subreddits related to psychiatric disorders: r/adhd, r/anxiety, r/bipolarreddit, r/bpd, r/depression, r/ptsd, and r/schizophrenia collected between December 2018 and December 2019. These subreddits were chosen to encompass a broad spectrum of psychiatric conditions. Each post was examined for the presence of regular expressions directly related to the subreddit title, and any posts containing such references were excluded from the dataset (Table 1).

**Table 1.** Regex codes used to remove posts with potentially revealing words from within each subreddit.

Subreddit	Regex terms for cleaning
r/adhd	adhd|attention|hyperact
r/anxiety	anxiety
r/bipolarreddit	bipolar
r/bpd	borderline|bpd
r/depression	depress
r/pstd	ptsd|post-traumatic|post traumatic
r/schizophrenia	schiz

### Embedding Generation

**Figure 1.**
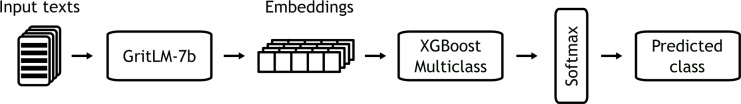
Posts from subreddits related to psychiatric disorders: r/adhd, r/anxiety, r/bipolarreddit, r/bpd, r/depression, r/ptsd, and r/schizophrenia collected between December 2018 and December 2019 are used as input into the GritLM-7B model to generate an embedding. Embeddings of all posts are used as features in a cross-validated XGBoost classifier to predict subreddit of origin. GritLM-7B: Generative Representational Instruction Tuning Language Model; XGBoost: Extreme Gradient Boosting.

We generated embeddings using the GritLM-7B model [9] (Figure 1). The GritLM-7B model is based on Mistral 7B [12] and fine-tuned using both representational instruction tuning and generative instruction tuning, resulting in a model that achieves state-of-the-art performance for both generative and embedding tasks. Representational instruction tuning enhances the model’s ability to understand and represent the underlying structure and semantics of the input data. This process involves training the model on tasks that require it to generate meaningful embeddings or representations of the input text, which can then be used for various downstream tasks such as classification, clustering, or retrieval. Conversely, generative instruction tuning aims to improve the model’s capability to generate coherent and contextually appropriate text based on given instructions or prompts. This involves training the model on tasks that require it to produce text outputs, such as summarization, translation, or creative writing. The overall training of the GritLM-7B model uses a loss function that is a weighted average of the loss functions for the individual representational and generative instructional tuning tasks, enabling the model to achieve a balanced proficiency in both comprehending and generating text.

In this study, we focus on the representational component of the GritLM-7B model. For embedding tasks, GritLM-7B uses bidirectional attention followed by mean pooling of the final hidden state to generate the final representation. The model inference was performed on a single GPU (NVIDIA A10G) Amazon Web Services EC2 instance.

### 2D Visualization of Embedding Space

We began by standardizing the embedding matrix to ensure that each feature had zero mean and unit variance. This standard scaling process is crucial for normalizing the data and mitigating the effects of differing scales among features. Following this, we used Uniform Manifold Approximation and Projection (UMAP) to reduce the dimensionality of the scaled data, transforming it into a 2D representation. UMAP is a powerful nonlinear dimensionality reduction technique that preserves the local and global structure of the data. For our UMAP transformation, we set the number of neighbors to 50 and the minimum distance parameter to 0.05, chosen by visual inspection to optimize the balance between local and global data structure preservation.

The number of neighbors parameter determines the size of the local neighborhood UMAP considers when learning the manifold structure of the data. A higher number of neighbors allows UMAP to capture more of the global structure of the data, ensuring that the overall shape and relationships between clusters are well-preserved. This is particularly important in our context, where understanding the relationships between different conditions may provide insights into which conditions have features of spontaneous communication that overlap with other conditions. The minimum distance parameter controls how tightly UMAP packs points together in the low-dimensional space. A smaller minimum distance value allows points to be closer together, which can help in preserving the local structure and making clusters more distinct. Decreasing this parameter enhances the separation between different clusters, making it easier to identify and interpret distinct groups within a label that may reflect the heterogeneity of a particular condition. Finally, we visualized the resulting 2D UMAP representation by creating a scatter plot, where each point was colored according to its corresponding label, facilitating the identification of patterns and clusters within the high-dimensional data.

### Classification Model

For the classification task, we used the Extreme Gradient Boosting (XGBoost) algorithm, using a multiclass classifier with a softmax objective function to predict the class labels for the posts from the 7 psychiatric disorder subreddits. Given that tree-based methods are not sensitive to the scale of the input features, we did not perform any standardization or normalization of the embeddings before using XGBoost. To address potential biases arising from class imbalance, we applied balanced class weighting, which assigns weights to each class that are inversely proportional to their frequencies. We configured the XGBoost classifier with the multiclass softmax objective function (multi:softmax) and retained the default parameter settings (eg, max_depth=6, learning_rate=0.3, n_estimators=100, booster=gbtree). No hyperparameter tuning was conducted. This approach ensured a straightforward implementation while leveraging the robust performance characteristics of the XGBoost algorithm for our multiclass classification task.

### Performance Evaluation

To evaluate the performance of the classification model, we used 10-fold cross-validation. We calculated class-wise precision, recall, and *F*_1_-scores to assess the performance of each psychiatric disorder–associated subreddit class. In addition, we computed macro and weighted average scores across all classes to provide a comprehensive evaluation of the model’s performance.

### Ethical Considerations

Our study strictly adheres to ethical guidelines for the use of internet-sourced data in research, ensuring that no harm comes to the individuals whose posts were analyzed.

This study used a publicly available dataset consisting of tens of thousands of posts from Reddit. The data were collected from forums that do not require registration or login to access and are therefore considered part of the public domain of the internet. In accordance with the journal’s policy, the analysis of large-scale publicly available online text data is not classified as human participants research and thus does not require institutional review board approval.

To further protect user privacy, all analyses were conducted at the aggregate level, and no attempts were made to identify or contact individual users. Usernames and any potentially identifying information were excluded from the analysis and presentation of results. The research was conducted in accordance with ethical standards for the use of online data, including respect for user anonymity and contextual integrity.

## Results

### Data Description

**Figure 2.**
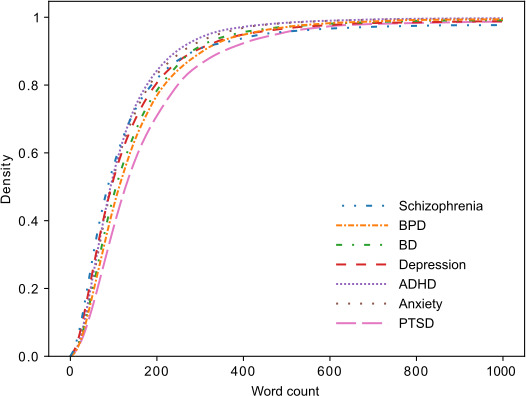
Cumulative distribution of word count in individual posts for each subreddit used in the study, with the word count axis cut off at 1000 words to better show the distribution at lower word counts. ADHD: attention-deficit/hyperactivity disorder; BD: bipolar disorder; BPD: borderline personality disorder; PTSD: posttraumatic stress disorder.

The data used in this study comes from seven distinct subreddits: r/adhd, r/anxiety, r/bipolarreddit, r/bpd, r/depression, r/ptsd, and r/schizophrenia. Following the removal of posts containing text that would be revealing of the subreddit (see “Methods” section), there was a nearly 7-fold difference between the total number of posts in each subreddit. The r/depression subreddit had the greatest number of posts (11,513 posts, 11,483 unique users) and the r/bipolarreddit had the least number of posts (1711 posts, 1633 unique users) (Table 2). Overall, 36,102 out of 37,195 (97.1%) posts were made by unique users. Among the users, 2 made 5 posts, 7 made 4 posts, and 54 made 3 posts. The remaining 36,039 (99.8%) made only 1 or 2 posts. No user made posts in more than one subreddit. All subreddits had mean and median lengths of approximately 150 words (Figure 2).

**Table 2.** Number of posts, unique users, and mean post length for each subreddit following post cleaning.

Subreddit	Posts, n	Unique users, n	Post length (words), mean (SD)
r/adhd	7568	7319	121.5 (106.2)
r/anxiety	6391	5990	126.4 (117.9)
r/bipolarredit	1711	1633	138.6 (124.9)
r/bpd	5849	5699	147.9 (132.6)
r/depression	11,513	11,483	132.2 (160.5)
r/ptsd	1954	1769	167.6 (158.0)
r/schizophrenia	2209	2209	123.4 (147.9)

### Relationships Between Categories

**Figure 3.**
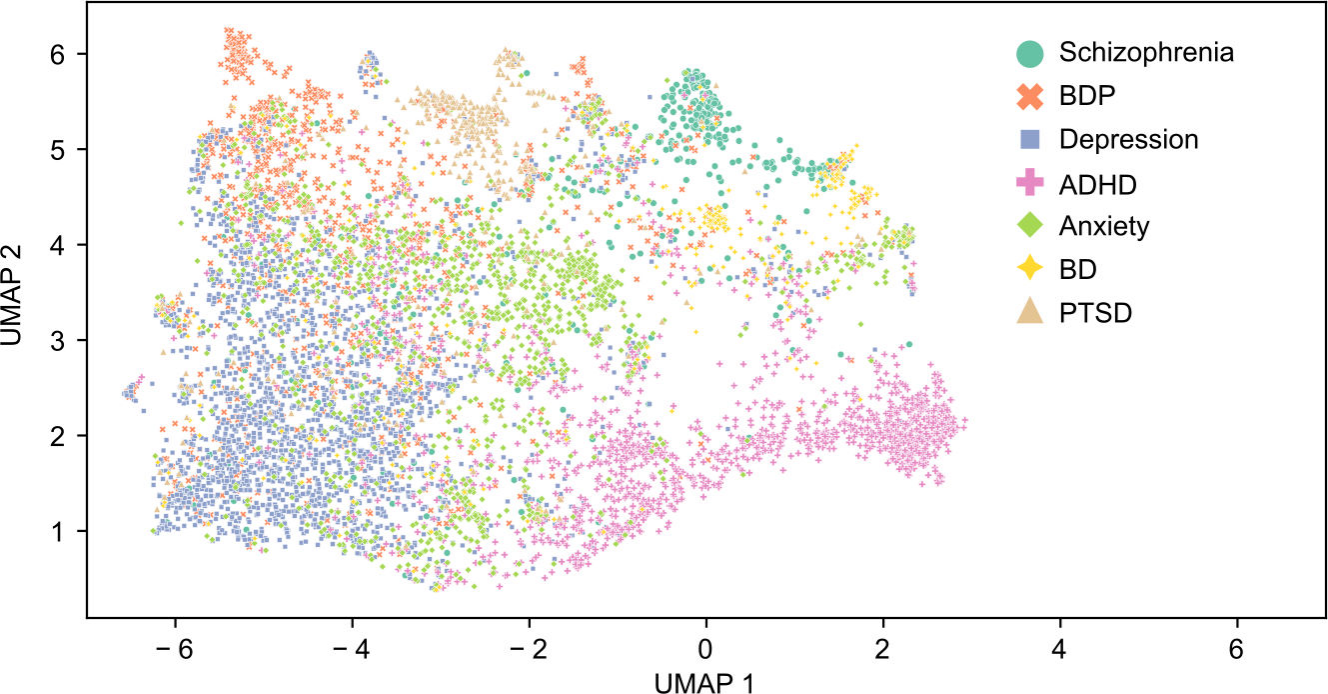
2D UMAP of GritLM-7B embeddings of every fifth Reddit post, where each dot represents an individual post. ADHD: attention-deficit/hyperactivity disorder; BD: bipolar disorder; BPD: borderline personality disorder; GritLM-7B: Generative Representational Instruction Tuning Language Model; PTSD: posttraumatic stress disorder; UMAP: Uniform Manifold Approximation and Projection.

We used the UMAP algorithm to generate a 2D representation of the embedding space facilitating the visualization of relationships between subreddits (Figure 3). This low-dimensional visualization reveals several qualitative features of the dataset. Notably, posts from r/anxiety subreddit are centrally located in the projection, adjacent to all other categories. This central positioning may indicate that the language used in discussions about anxiety is present across all subreddits. In addition, while some subreddits (r/ptsd, r/bipolarredit, r/adhd, and r/schizophrenia) have distinct clusters, the other 3 subreddits (r/anxiety, r/bpd, and r/depression) have more overlapping point clouds, suggesting greater linguistic similarity across the latter 3 subreddits.

**Figure 4.**
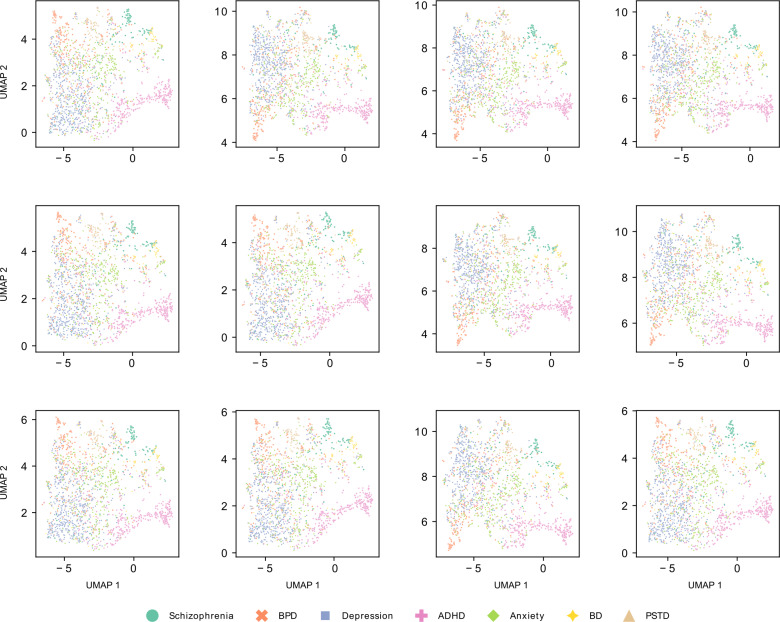
Additional projections of the GritLM-7B embedding data from the UMAP algorithm using the same parameters as in Figure 3, where each plot is generated by using a different random seed. ADHD: attention-deficit/hyperactivity disorder; BD: bipolar disorder; BPD: borderline personality disorder; GritLM-7B: Generative Representational Instruction Tuning Language Model; PTSD: posttraumatic stress disorder; UMAP: uniform manifold approximation and projection.

**Figure 5.**
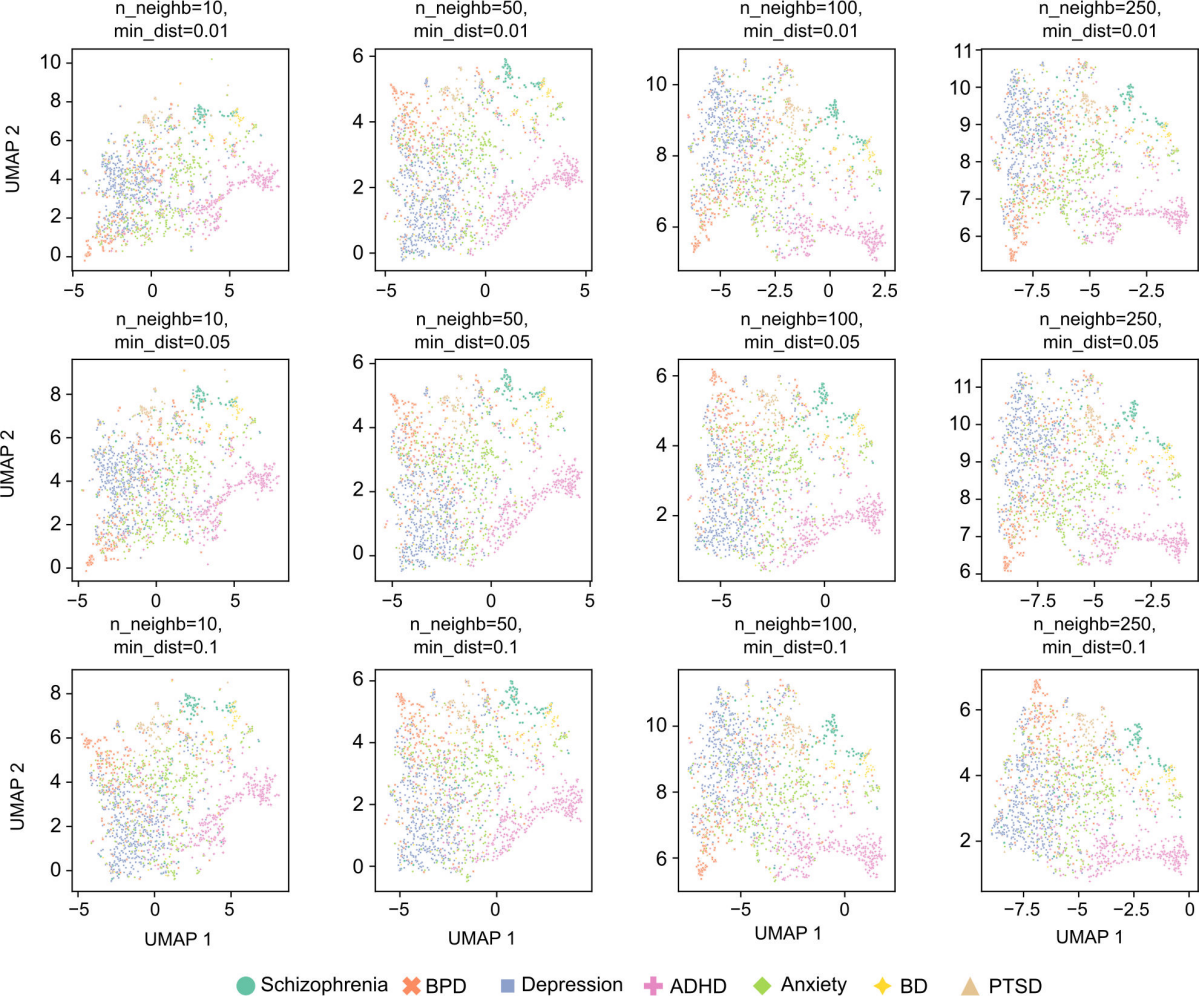
Additional projections of the GritLM-7B embedding data from the UMAP algorithm, varying the local neighborhood and minimum distance parameters. ADHD: attention-deficit/hyperactivity disorder; BD: bipolar disorder; BPD: borderline personality disorder; GritLM-7B: Generative Representational Instruction Tuning Language Model; PTSD: posttraumatic stress disorder; UMAP: Uniform Manifold Approximation and Projection.

The UMAP algorithm is inherently stochastic and can be sensitive to initial conditions. However, UMAP is designed to preserve the global structure of the data, making it more stable and less sensitive to parameter changes and initial conditions compared with other dimensionality reduction algorithms such as t-distributed Stochastic Neighbor Embedding. To illustrate this stability, we present UMAP projections using the specified parameters with different random seeds (Figure 4) and with the same random seed but varying local neighborhood and minimum distance parameters (Figure 5). In both cases, only every 20th post was plotted to allow for easier visualization of the distribution of the different categories. The resulting plots consistently support the qualitative observations described above.

### Classifier Performance

In addition to visualizing the embedding space of subreddit categories using UMAP, we used a 10-fold cross-validated XGBoost classifier to predict the subreddit of origin for each post. The support-weighted average precision, recall, and *F*_1_-scores across all categories were 0.73, 0.73, and 0.73, respectively. The macroaverage precision, recall, and *F*_1_-scores were 0.73, 0.68, and 0.7, respectively. Furthermore, the overall accuracy of the model was measured at 0.73.

At the individual category level, the model performed best on r/adhd (*F*_1_-score=0.86), r/depression (*F*_1_-score=0.77), worst on r/bipolarreddit (*F*_1_-score=0.63), and r/bpd (*F*_1_-score=0.58) (Table 3). These results indicate that the XGBoost classifier demonstrated moderate predictive performance in identifying the subreddit from which a post originated.

**Table 3.** Performance metrics of the Extreme Gradient Boosting multiclass classifier using Generative Representational Instruction Tuning Language Model (GritLM-7B) embeddings as features. Metrics are displayed for each individual subreddit, as well as the average performance across all subreddits, using a 0.5 classification threshold.

Subreddit	Precision	Recall	*F*_1_-score
r/adhd	0.86	0.86	0.86
r/anxiety	0.66	0.69	0.67
r/bipolarredit	0.68	0.5	0.58
r/bpd	0.65	0.61	0.63
r/depression	0.73	0.81	0.77
r/ptsd	0.78	0.63	0.7
r/schizophrenia	0.76	0.62	0.69
Macroaverage	0.73	0.68	0.7
Weighted average	0.73	0.73	0.73

**Figure 6.**
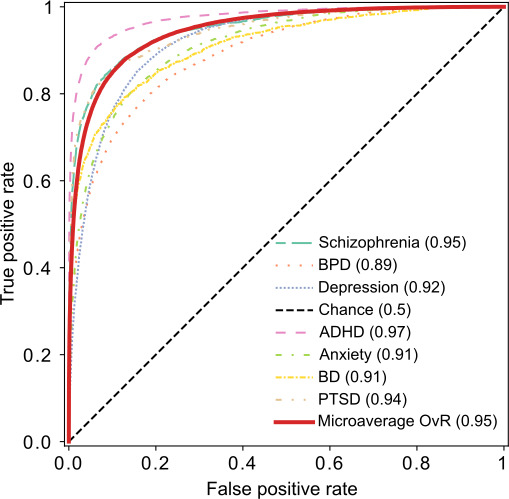
Receiver operating characteristic curve and area under the receiver operating characteristic curve (AUC) values for a one-versus-rest (OvR) Extreme Gradient Boosting (XGBoost) classifier using GritLM-7B embeddings, shown for each subreddit against all other classes. ADHD: attention-deficit/hyperactivity disorder; BD: bipolar disorder; BPD: borderline personality disorder; GritLM-7B: Generative Representational Instruction Tuning Language Model; PTSD: posttraumatic stress disorder.

For each subreddit, we used the classifier probabilities to estimate the AUC for a one-versus-rest classification task (Figure 6). The AUC values were notably high, ranging from 0.89 to 0.97, indicating that posts within each subreddit are highly distinguishable from the other subreddits. The r/adhd subreddit had the highest AUC (0.97), suggesting that this topic is most distinct from other topics. Conversely, the r/bpd subreddit post had the lowest AUC (0.89) indicating that its posts may share more linguistic features with those of other subreddits.

**Figure 7.**
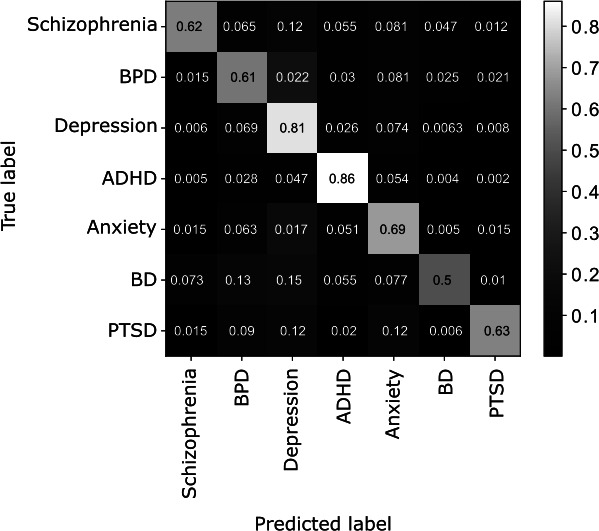
Confusion matrix from a multiclass Extreme Gradient Boosting classifier using GritLM-7B embeddings to classify posts into subreddits related to mental health conditions. ADHD: attention-deficit/hyperactivity disorder; BD: bipolar disorder; BPD: borderline personality disorder; GritLM-7B: Generative Representational Instruction Tuning Language Model; PTSD: posttraumatic stress disorder.

We computed a confusion matrix to assess the performance of the multiclass classifier at both the individual category level and across pairs of categories (Figure 7). This analysis enables us to examine the misclassification rates between different categories and gain insights into the specific types of errors made by the classifier. The four most common true-predicted category confusions were identified as follows: r/bpd-r/depression, r/anxiety-r/depression, r/bipolarreddit-r/depression, and r/bipolarreddit-r/bpd. Notably, the model frequently misclassifies posts as originating from r/depression. This may result from users in r/bpd, r/anxiety, and r/bipolarreddit using language that is more similar to that used by users in r/depression. Interestingly, the confusion rates between r/anxiety, r/bpd, and r/bipolarreddit are less than 0.13, suggesting that linguistic overlap between these subreddits is less pronounced with each other than they each are with r/depression. In addition, this result may be partially explained by the overrepresentation of the r/depression class in the dataset that is incompletely offset by class weighting when training the model.

Finally, we compared the performance of GritLM-7B embeddings with other state-of-the-art methods for generating sentence embeddings, specifically OpenAI’s text-embedding-3-small [13] and Sentence-Bidirectional Encoder Representations from Transformers (S-BERT) [14]. Across all evaluation metrics, the classifier using GritLM-7B embeddings outperformed those using OpenAI and S-BERT embeddings (Table 4). These results are consistent with previous findings that GritLM-7B achieves state-of-the-art performance on embedding-based tasks.

**Table 4.** Performance comparison of multiclass Extreme Gradient Boosting classifiers for categorizing posts into mental health–related subreddits, using embeddings from large language models (Generative Representational Instruction Tuning Language Model [GritLM-7B] and OpenAI) and S-BERT^a^.

Large language models	Classifier performance		Weighted average		
	Accuracy	AUC^b^	Precision	Recall	*F*_1_-score
GritLM-7B	0.73	0.95	0.73	0.73	0.73
OpenAI	0.7	0.94	0.7	0.7	0.7
S-BERT	0.54	0.86	0.47	0.45	0.46

a S-BERT: sentence-bidirectional encoder representations from transformers

b AUC: area under the receiver operating characteristic curve

## Discussion

### Principal Findings

The use of LLMs offer a novel approach to analyzing patterns of language usage from spontaneous, patient-generated communication. In the field of psychiatric disorders, this has the potential to revolutionize the way we diagnose and monitor these conditions. By analyzing the spontaneous use of language in online discussion data, we can gain valuable insights into the linguistic patterns that distinguish between different psychiatric disorders. In this study, we used embeddings derived from the GritLM-7B LLM to classify posts originating from subreddits dedicated to seven common conditions: schizophrenia, BPD, depression, ADHD, anxiety, posttraumatic stress disorder, and BD. Our work provides a proof of concept showing that modern LLMs can be effectively used to differentiate between spontaneous communication related to different psychiatric disorders. This novel application demonstrates the potential of LLMs to identify distinct linguistic markers associated with various mental health conditions, paving the way for innovative diagnostic and monitoring tools in psychiatric care.

We found that the XGBoost classifier using features generated from GritLM-7B embeddings exhibits high, though not perfect, performance. Symptoms of psychiatric disorders span a number of different modalities, such as sleep, appetite, and motor activity [15-17]. Therefore, in addition to standard reasons for misclassification such as overfitting and noisy or limited data, posts may be misclassified due to similarities in presentations across the different psychiatric disorders, or high rates of comorbidity between disorders. In our study, the pair-wise misclassification rates may reveal disorders where the presentation of symptoms that are likely to be revealed by spontaneous speech are more similar. For example, posts coming from r/bpd are likely to be misclassified as coming from r/depression. This is consistent with previous studies showing similarities in word usage [18] and high rates of comorbidity for BD and depression [19].

We observe a range of performances in the one-versus-rest classifiers across different disorders. Notably, BPD has the lowest AUC (0.89). This may be because BPD shares many symptoms with other conditions and is often comorbid with them [20]. A key differentiating factor between BPD and related conditions is the temporal nature of symptoms. In BPD, core symptoms are typically more variable and transient compared with their presentation in related conditions. The dataset used in this study consists mostly of single social media posts from each user, providing a cross-sectional view rather than a longitudinal one. This cross-sectional data does not capture within-subject symptom variability which may explain the reduced performance of the BPD classifier. In contrast, ADHD has the highest AUC (0.97). This could be because ADHD is a neurodevelopmental disorder, unlike the other conditions examined in this work. As a result, spontaneous communication related to ADHD may be more distinct from other conditions, leading to higher classifier performance.

### Limitations

One limitation of analyzing posts from mental health disorder subreddits is that they may not necessarily originate from officially diagnosed patients. The individuals participating in these online communities may self-identify with a particular disorder without having received a formal diagnosis from a health care professional or maybe caregivers supporting a diagnosed individual. As a result, the content may not be directly clinically relevant. Furthermore, psychiatric disorders have high rates of comorbidity [21,22]. Posts within a single subreddit may come from an individual with multiple psychiatric pathologies, limiting classification into any one specific category. Finally, psychiatric disorders exist on a spectrum [23,24] and can have multiple subtypes [25,26], which may not be well-captured in a classification framework. Therefore, the insights gained from analyzing these posts should be interpreted with caution and may not fully represent the experiences and perspectives of clinically diagnosed individuals. However, future research could apply this methodology to free speech data generated by verified patients and leverage more precise clinical diagnostic labels, diagnostic history, and finer-tuned model predictions (eg, predicting subtype or severity) to derive further clinical findings.

In addition, since subreddits posts are unprompted, the content of the posts, in addition to the syntax and semantics may vary across the different subreddits. For instance, r/ptsd posts may be more likely to have references to traumatic events, while r/adhd posts may reference learning challenges more often. Therefore, the embedding space may separate the different categories by variations in the topics or themes across subreddits, in addition to features related to the way language is used in the posts. Nevertheless, this could still hold clinical significance as specific themes, like paranoia in schizophrenia or anhedonia in depression, have diagnostic relevance.

Embeddings used by LLMs are black-box numeric representations of the input text, making it difficult to interpret the features that are being used to separate classes and potentially limiting the usefulness and adoption in clinical applications. However, novel methods have shown that generative models may be useful for supporting interpretable embeddings [27]. In addition to state-of-the-art performance on representational tasks, GritLM-7B also performs highly on generative tasks. Thus, in combination with feature explanation, GritLM-7B may be better suited than models optimized for only representational tasks for leveraging generative approaches to interpreting clinically relevant features.

### Conclusions

This study demonstrates the potential of using LLMs to analyze free speech for the diagnosis and monitoring of psychiatric disorders. The results suggest that LLMs can provide valuable insights into the linguistic patterns that differentiate various psychiatric conditions. These patterns can be leveraged to develop more objective, efficient, and patient-centered strategies for assessment, monitoring, and research. However, further research is needed to validate these findings in a well-defined clinical population and explore the limitations of this approach.

## Abbreviations

ADHD: attention-deficit/hyperactivity disorder
AUC: area under the receiver operating characteristic curve
BD: bipolar disorder
BPD: borderline personality disorder
GAD-7: Generalized Anxiety Disorder 7-item
GritLM-7B: Generative Representational Instruction Tuning Language Model
LLM: large language model
PTSD: posttraumatic stress disorder
S-BERT: sentence-bidirectional encoder representations from transformers
UMAP: Uniform Manifold Approximation and Projection
XGBoost: Extreme Gradient Boosting
